# Stem Cells as Therapy for Necrotizing Enterocolitis: A Systematic Review and Meta-Analysis of Preclinical Studies

**DOI:** 10.3389/fped.2020.578984

**Published:** 2020-12-09

**Authors:** Eduardo Villamor-Martinez, Tamara Hundscheid, Boris W. Kramer, Carlijn R Hooijmans, Eduardo Villamor

**Affiliations:** ^1^Department of Pediatrics, Maastricht University Medical Center (MUMC+), School for Oncology and Developmental Biology (GROW), Maastricht, Netherlands; ^2^Department for Health Evidence Unit SYRCLE, Radboud University Medical Center, Nijmegen, Netherlands

**Keywords:** stem cells, necrotizing enterocolitis (NEC), preclinical (*in-vivo*) studies, meta-analysis, risk of bias assessment

## Abstract

**Background:** Necrotizing enterocolitis (NEC) is the most common life-threatening gastrointestinal condition among very and extremely preterm infants. Stem cell therapy has shown some promising protective effects in animal models of intestinal injury, including NEC, but no systematic review has yet evaluated the preclinical evidence of stem cell therapy for NEC prevention or treatment.

**Methods:** PubMed and EMBASE databases were searched for studies using an animal model of NEC with stem cells or their products. The SYRCLE tool was used for the assessment of risk of bias. A random-effects model was used to pool odds ratios (ORs) and 95% confidence interval (CI).

**Results:** We screened 953 studies, of which nine (eight rat and one mouse models) met the inclusion criteria. All animal models induced NEC by a combination of hypothermia, hypoxia, and formula feeding. Risk of bias was evaluated as unclear on most items for all studies included. Meta-analysis found that both mesenchymal and neural stem cells and stem cell-derived exosomes reduced the incidence of all NEC (OR 0.22, 95% CI 0.16–0.32, *k* = 16), grade 2 NEC (OR 0.41, 95% CI 0.24–0.70, *k* = 16), and grade 3–4 NEC (OR 0.28, 95% CI 0.19–0.42, *k* = 16). *k* represents the number of independent effect sizes included in each meta-analysis. The effect of the exosomes was similar to that of the stem cells. Stem cells and exosomes also improved 4-day survival (OR 2.89 95% CI 2.07–4.04, *k* = 9) and 7-day survival (OR 3.96 95% CI 2.39–6.55, *k* = 5) after experimental NEC. Meta-analysis also found that stem cells reduced other indicators of intestinal injury.

**Conclusion:** The data from this meta-analysis suggest that both stem cells and stem cell-derived exosomes prevented NEC in rodent experimental models. However, unclear risk of bias and incomplete reporting underline that poor reporting standards are common and hamper the reliable interpretation of preclinical evidence for stem cell therapy for NEC.

## Introduction

Necrotizing enterocolitis (NEC) is the most common life-threatening gastrointestinal condition among very and extremely preterm infants (1–4). Severe NEC is characterized by full-thickness destruction of the intestine leading to intestinal perforation, peritonitis, bacterial invasion of blood stream, and generalized infection (1–4). NEC has a multifactorial etiology that is largely related to prematurity and the consequent immaturity of the gastrointestinal tract (1–10). However, besides low gestational age (GA), several risk factors such as formula feeds, gut dysbiosis, infection, or intestinal hypoperfusion have been implicated in the etiopathogenesis of NEC (1–10). Furthermore, a clear definition of NEC remains elusive because NEC likely represents different conditions with intestinal injury or necrosis as final outcome (4, 11).

A number of animal models have been developed to investigate the pathophysiology of NEC and to test different preventive and therapeutic strategies (12–18). The most widely used experimental animals are rats, mice, and piglets (12–18). The experimental models try to reproduce the pathogenesis of NEC using one or a combination of potential contributory factors such as intestinal immaturity, formula feeding, bacteria and/or their byproduct, and hypoxic–ischemic stress (12–18). However, after more than five decades of preclinical and clinical research on NEC, the promise of a “magic bullet” for the prevention and/or treatment of NEC has yet to become a reality (11).

Stem cell therapy is increasingly proposed as a novel therapeutic approach for a number of complications of prematurity such as bronchopulmonary dysplasia (19–22), brain injury (22, 23), or retinopathy of prematurity (24) with encouraging preclinical results auspicious for clinical translation (19, 21, 22). A growing number of preclinical studies have investigated the potential therapeutic role of stem cells in experimental NEC (13–21). However, to use stem cell therapy, we need to answer five questions: which cells to give, at what time, via which route, in which dose, and to which babies (25).

Systematic reviews and meta-analyses are common practice in human clinical research, particularly for randomized controlled trials. Although most animal experiments are performed to enrich our clinical understanding of human diseases, systematic reviews of preclinical studies are still scarce (26, 27). Meta-analyses of preclinical studies can be used to inform clinical trial design, to understand the possible discrepancies between findings from preclinical and clinical studies and to improve the quality and translational utility of animal experimentation (28–30). To date, there has been no systematic review and/or meta-analysis on the therapeutic potential of stem cells in experimental NEC. We aimed to provide a comprehensive overview of all studies using stem cells in animal models of NEC and to quantify the effects of stem cells in mortality and development of intestinal injury.

## Methods

### Protocol

A protocol was registered on PROSPERO (CRD42018110084) before starting the review (31). We used the Preferred Reporting Items for Systematic Reviews and Meta-Analyses (PRISMA) checklist for the manuscript (32).

### Sources and Search Strategy

We searched PubMed and EMBASE databases for original, preclinical studies concerning the effects of stem cells or stem cell products on NEC published until June 2019. The search strategy involved the following four search components: stem cells (and all synonyms) AND (necrotizing enterocolitis OR [neonate AND (intestinal injury OR necrosis)] AND animals (33, 34) (for our complete search strategy, see S 1). No language or date restrictions were applied. Electronic search alerts were set up using Google Scholar to be able to include any studies published after the last search.

### Inclusion Criteria and Study Selection

Studies were included in this systematic review when they met all of the following criteria, defined *a priori* in our published protocol: (1) original *in vivo* animal studies using a neonatal experimental NEC model; (2) intervention was randomized, quasi-randomized, or non-randomized; (3) tested as intervention stem cells or stem cell-derived products; and (4) reported on any of the following outcome measures: survival, NEC incidence and/or severity, intestinal injury, weight gain, and clinical sickness scores. Non-interventional studies, studies without controls, and non-neonatal models of intestinal injury were excluded.

The primary outcome was NEC incidence. Secondary outcomes were survival and severity of NEC. Therefore, studies that reported on survival, NEC incidence (determined histologically), and/or severity of NEC (determined histologically and with predetermined scales) were included.

Two independent reviewers (TH and EV-M) screened all studies for inclusion. The first screening was based on title and abstract using Rayyan RCI software (35). In case of doubt, the full text of the article was evaluated. The full text of all preselected publications was subsequently assessed by two independent reviewers (TH and EV-M). Studies were included when they met all the inclusion criteria. Disagreements about inclusion were resolved through discussion and consensus among three reviewers (TH, EV-M, and CH).

One reviewer (TH) extracted the data using a predetermined data extraction sheet. A second reviewer (EV-M) checked data for accuracy. Data were extracted for study characteristics (authors, year of publication, and study location), study design (sample size for intervention, control, and sham groups), intervention characteristics (timing, dose, and mode of stem cell administration), and outcome measures (primary and secondary outcomes as described above). Dichotomous and continuous data provided in numbers were extracted directly. Data on survival and NEC incidence/severity were converted to odds ratios (ORs), and data on intestinal injury (intestinal permeability and motility), weight, and sickness/severity scores were converted to standardized differences in means. If only graphs were available, Web Plot Digitizer was used to extract numerical values (36).

### Risk of Bias

We used the SYRCLE tool (37) to assess the risk of bias in the included studies. Two reviewers (TH and EV-M) independently evaluated the studies, and discrepancies in scoring were resolved through discussion. A “yes” score indicates low risk of bias; a “no” score indicates high risk of bias; and a “?” score indicates unknown risk of bias.

In order to avoid the problem of too many items being evaluated as “unclear risk of bias” due to lack of reporting of experimental details on animals, methods, and materials (38), we added three items on reporting and evaluated whether studies reported (1) any measure of randomization, (2) any measure of blinding, and (3) a sample size calculation. For these three items, a “yes” score indicates “reported,” and a “no” score indicates “unreported.”

### Meta-Analysis, Subgroup Analyses, and Publication Bias

Studies were combined and analyzed using Comprehensive Meta-Analysis V3.0 software (Biostat Inc., Englewood, NJ, USA). The unit of analysis for the meta-analysis (represented as *k*) was the individual experiments (i.e., one reference could contain multiple independent experiments). All outcome measures that were reported in more than one study were included in meta-analyses. For dichotomous outcomes, the OR with 95% confidence interval (CI) was calculated from the data provided in the studies. For continuous outcomes, we extracted the mean, SD, and n to calculate the standardized mean difference (SMD). If studies used a single control group and multiple experimental groups, we corrected for this by dividing the sample size of the control group by the number of experimental groups. When one of the cells contained a zero value or the risk in either the control or experimental group was 100%, we added 0.5 to each cell to calculate the OR.

Due to anticipated heterogeneity, summary statistics were calculated with a random-effects model. This model accounts for variability between studies as well as within studies. Statistical heterogeneity was assessed by Cochran's Q statistic and by the I^2^ statistic, which is derived from Q and describes the proportion of total variation that is due to heterogeneity beyond chance (39).

Subgroup analyses were predefined in the protocol and only carried out if there were at least three independent comparisons per subgroup (*k* ≥ 3). The criteria we planned on using for subgroup analyses included cell-type, animal/species, stem cells vs. cell-derived products, control group (placebo/sham vs. no intervention), and definition of neonatal (term vs. preterm model). Subgroup analyses were conducted according to the mixed-effects model (40). In this model, a random-effects model is used to combine studies within each subgroup, and a fixed-effects model is used to combine subgroups and yield the overall effect. We expected the variance to be comparable within the subgroups; therefore, we assumed a common among-study variance across subgroups. For subgroup analyses, we adjusted our significance level according to the conservative Bonferroni method to account for multiple analyses (p/number of comparisons).

Publication bias analysis was carried out for the outcomes with *k* > 10. We used visual inspection of the funnel plot, Duval and Tweedie's trim and fill, and Egger's regression test to assess publication bias (40).

## Results

### Study Characteristics and Experimental Models

**Figure 1:**
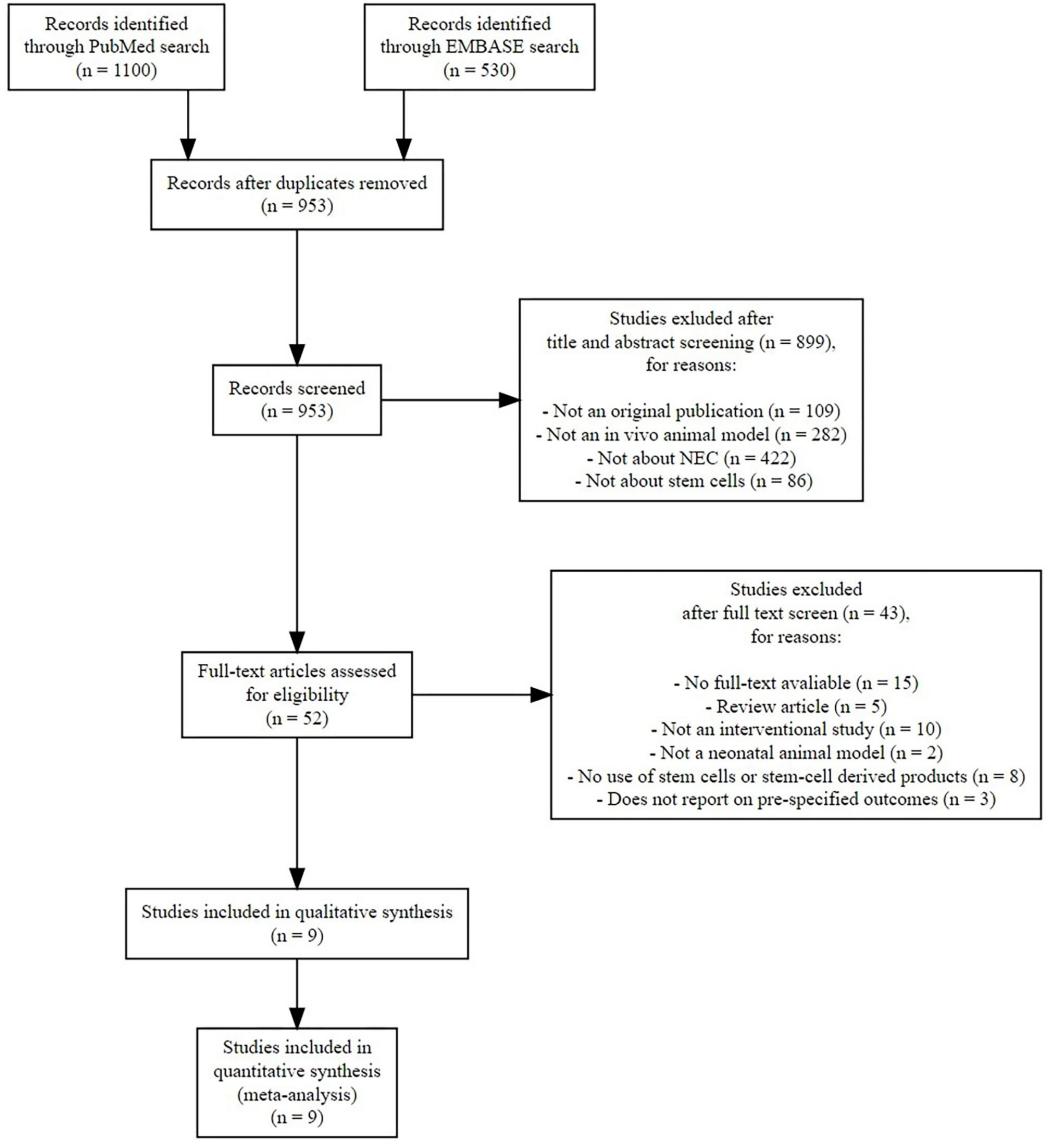
Preferred Reporting Items for Systematic Reviews and Meta-Analyses (PRISMA) flow diagram of systematic search.

After duplicates were removed, 953 references needed to be screened for eligibility. Figure 1 shows the PRISMA diagram of the comprehensive search and the reasons for excluding studies. Finally, nine studies could be included in this systematic review (13–21), which encompassed 23 comparisons in total. We did not find additional studies after the search through electronic alerts (until December 2019). Six of the included studies (41–46) were carried out in the same research facility in Columbus, Ohio (USA). One study (47) was carried out in Turkey, and two studies were carried out in the United Kingdom (48, 49). The characteristics of included studies are summarized in Table 1. Eight studies used rats, and the study of Wei et al. used mice. All included studies used the method of Barlow et al. (50) for inducing experimental NEC through formula feeding, hypoxia, and hypothermia.

**Table 1:** Summary of the characteristics of studies in each article.

**Author**	**Species**	**INT**	**CON**	**No. INT**	**No. CON**	**Dose and time of intervention**	**Admin mode**	**NEC induced at age**	**Outcome measures**
McCulloh et al. (42)	Rat	AFMSC BMMSC ENSC AFNSC	PBS	42 48 36 37	62	2 ^*^ 10^6^ cells in 0.1 ml of PBS after delivery	IP	Preterm (−0.5 day)	NEC incidence, NEC severity
McCulloh et al. (41) Series A	Rat	AFMSC Exo BMMSC Exo ENSC Exo AFNSC Exo	PBS	18 15 9 14	14	8 ^*^ 10^7^ exosomes < 1 h after birth	IP	Preterm (−1 day), immediately after intervention	NEC incidence, NEC severity
McCulloh et al. (41) Series B	Rat	AFMSC Exo BMMSC Exo ENSC Exo	PBS	20 13 11	14	4 ^*^ 10^8^ exosomes < 1 h after birth	IP	Preterm (−1 day), immediately after intervention	NEC incidence, NEC severity
Rager et al. (43)	Rat	BMMSC BMMSC Exo	PBS	35 40	46	Cells in 50 ml of PBS, 5 h after delivery	IP	Preterm (−1 day)	NEC incidence, NEC severity, intestinal injury
Tayman et al. (47)	Rat	BMMSC	None	12	12	6 ^*^ 10^5^ cells in 50 μl of PBS, 3rd day of study	IP	Term (0 days)	Survival, sickness score
Wei et al. (44)	Mouse	ENSC	HBSS	39	48	30 μl of HBSS, 2 h after birth	IP	Term (0 days)	NEC incidence, NEC severity, intestinal injury
Yang et al. (45)	Rat	BMMSC IP BMMSC IV	Vehicle only	25 27	38	300 ^*^ 10^3^ in 40 μl of vehicle, at birth	IP IV	Preterm (−0.5 day)	Survival, NEC incidence, NEC severity, intestinal injury
Zani et al. (49) (*EJPS*)	Rat	AFS	PBS	92	93	24 h after birth	IP	Unknown	Survival
Zani et al. (48) (*Gut*) Series A	Rat	BMMSC	PBS	26	30	2 ^*^ 10^6^ cells in 50 μl of PBS, 24 h after birth	IP	Unknown	Survival, NEC severity, intestinal injury, sickness score
Zani et al. (48) (*Gut*) Series B	Rat	BMMSC AFS	PBS	17 40	43	2 ^*^ 10^6^ cells in 50 μl of PBS, 24 h after birth	IP	Unknown	Survival, NEC severity, intestinal injury, sickness score
Zani et al. (48) (*Gut*) Series C	Rat	AFS	PBS	121	120	2 ^*^ 10^6^ cells in 50 μl of PBS, 24 h after birth	IP	Unknown	Survival, NEC severity, intestinal injury, sickness score
Zhou et al. (46)	Rat	ENSC	DMEM/F12	9	9	50,000 cells in 50 μl of DMEM, day 3 after birth	IP	Preterm (−1 day)	Survival, intestinal injury

*INT, interventional group; CON, control group; Admin, administration; NEC, necrotizing enterocolitis; AF, amniotic fluid; MSC, mesenchymal stem cell; IP, intraperitoneal; exo, exosomes (stem cell product); IV, intravenous; PBS, phosphate-buffered saline; DMEM, Dulbecco's modified Eagle's medium; AFS, amniotic fluid-derived stem cells (stained positively for a number of surface markers characteristic of mesenchymal and/or neural stem cells); AFMSC, amniotic fluid mesenchymal stem cell; AFNSC, amniotic fluid neural stem cell; BMMSC, bone marrow mesenchymal stem cell; ENSC, enteric neural stem cell; HBSS, Hank's Balanced Salt Solution*.

### Intervention: Stem Cells and Stem Cell-Derived Products

As shown in Table 1, seven studies examined stem cells as the intervention (42, 44–49), one study examined both stem cells and exosomes (43), and one study examined only exosomes as intervention (41). Regarding the type of stem cells, one study (one experiment) (42) examined amniotic fluid (AF) mesenchymal stem cells (MSCs), and two studies (three experiments) (48, 49) examined AF stem cells (AFSCs). AFSCs stained positively for a number of surface markers that are characteristic of MSCs and/or neural stem cells (NSCs) (51, 52) and therefore were analyzed separately from AFMSCs. Five studies (seven experiments) (42, 43, 45, 47, 48) examined bone marrow MSCs (BMMSCs), three studies (three experiments) (42, 44, 46) examined enteric NSCs (ENSCs), and one study (one experiment) (42) examined AFNSCs. Two studies studied exosomes of stem cells as the intervention, with McCulloh et al. (41) studying exosomes of AFMSCs, BMMSCs, ENSCs, and AFNSCs in two experiments, and Rager et al. (43) studying BMMSC exosomes in one experiment. The dosages and timing for each intervention are detailed in Table 1.

### Reported Outcomes

#### Primary Outcomes

Four studies (16 experiments) (41–43, 45) reported on incidence of any grade NEC, incidence of grade 2 NEC, and incidence of severe NEC (grades 3 and 4). Five studies (nine experiments) reported on survival at day 4 of life (45–49), and two studies (five experiments) reported on survival at day 7 of life (46, 48).

#### Secondary Outcomes

Several studies reported on indicators of intestinal injury. Four studies (10 experiments) (42, 43, 45, 48) reported on intestinal permeability. Two studies (two experiments) reported on intestinal motility (46, 49). Two studies (two experiments) reported on clinical sickness (47, 48). One study (one experiment) reported on NEC severity score (48).

### Risk of Bias Assessment

#### Selection Bias and Performance Bias

The details for the risk of bias analysis are shown in Table 2. None of nine included studies provided sufficient information to assess the risk of bias as either low or high risk of bias for selection bias (*sequence generation, baseline characteristics*, and *allocation concealment*) and performance bias (*random housing* and *investigator/caregiver blinding*). We evaluated all six of these items as “unclear” risk of bias due to poor reporting of essential experimental details.

**Table 2:** Risk of bias assessment.

**Study**	**Adequate sequence generation**	**Baseline characteristics comparable**	**Allocation concealment**	**Random housing**	**Investigators/caregivers blinding**	**Random outcome assessment**	**Blinding outcome assessment**	**Incomplete outcome data adequate addressed**	**Free of reporting bias**	**Other biases absent**	**Randomization mentioned?**	**Blinding mentioned?**	**Sample size and calculation mentioned?**
McCulloh et al. (42)	?	?	?	?	?	?	Yes	?	?	?	Yes	Yes	No
McCulloh et al. (42)	?	?	?	?	?	?	Yes	No	?	?	Yes	Yes	No
Rager et al. (43)	?	?	?	?	?	?	?	?	?	Yes	Yes	No	No
Tayman et al. (47)	?	?	?	?	?	?	Yes	Yes	?	Yes	No	Yes	Yes
Wei et al. (44)	?	?	?	?	?	?	Yes	?	?	Yes	Yes	Yes	No
Yang et al. (45)	?	?	?	?	?	?	Yes	?	?	Yes	Yes	Yes	No
Zani et al. (49) (*EJPS*)	?	?	?	?	?	?	?	?	?	Yes	Yes	Yes	No
Zani et al. (48) (*Gut*)	?	?	?	?	?	?	Yes	?	No	Yes	Yes	Yes	No
Zhou et al. (46)	?	?	?	?	?	?	?	?	?	Yes	No	No	No

#### Detection Bias

We evaluated risk of bias for *blinding of outcome assessors* (detection bias) separately for each outcome reported, and the details of this evaluation are provided in Table 3. Five studies (41, 42, 44, 45, 47) had low risk of bias for *blinding of outcome assessors*, for the outcome NEC incidence and severity. One study (48) blinded outcome assessors for intestinal motility and scored “low” risk of bias for this outcome, whereas another (46) did not clarify if outcome assessors were blinded and scored “unclear” risk of bias. For all other outcomes (survival, intestinal permeability, and clinical sickness score), studies did not clarify if outcome assessors were blinded, and they were evaluated as “unclear” risk of bias. No study provided information on *random outcome assessment* (detection bias), and we evaluated this for all studies as “unclear” risk of bias.

**Table 3:** Risk of bias for blinding of outcome assessment, per outcome.

	**Was outcome assessment blinded?**		
**Study**	**Yes**	**Unclear**	**No**
McCulloh et al. (42)	NEC incidence	-	–
McCulloh et al. (42)	NEC incidence	–	–
Rager et al. (43)	–	NEC incidence, intestinal permeability	–
Tayman et al. (47)	NEC incidence, sickness score	Survival, clinical sickness score	–
Wei et al. (44)	NEC incidence	Intestinal permeability	–
Yang et al. (45)	NEC incidence	Survival, intestinal permeability	–
Zani et al. (49) (*EJPS*)		Survival	–
Zani et al. (48) (*Gut*)	Intestinal motility, intestinal permeability	Survival, clinical sickness score	–
Zhou et al. (46)	–	Survival, intestinal motility	–

*NEC, necrotizing enterocolitis*.

#### Attrition Bias

Attrition bias (incomplete outcome data) was evaluated as “low” in one study (44), “high” in another study (41), and “unclear” in seven studies (42, 43, 45–49).

#### Reporting Bias and Other Bias

Reporting bias (selective outcome reporting) was evaluated as “high” in one study (48) and unclear in eight studies (41–47, 49).

We evaluated seven out of nine studies as low risk of “other biases”: there were no indications of unit of analysis errors, and they reported information on funding and conflicts of interest. Two studies did not provide funding and conflict of interest information and were evaluated as “unclear” risk of bias in this category.

### Meta-Analyses of Primary Outcomes

#### Necrotizing Enterocolitis Incidence

**Figure 2:**
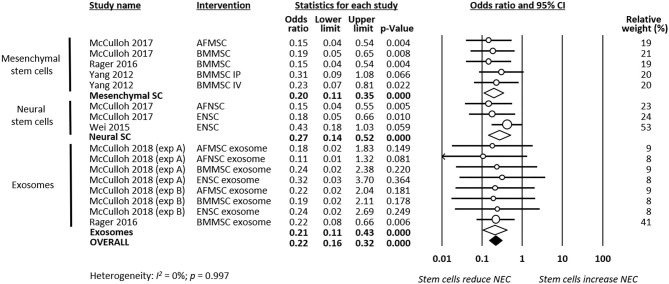
Meta-analysis on stem cells/stem cell-derived products and incidence of any grade necrotizing enterocolitis (NEC). AF, amniotic fluid; BM, bone marrow; ENSC, enteric neural stem cell; IP, intraperitoneal; IV, intravenous; MSC, mesenchymal stem cell; NSC, neural stem cell; SC, stem cell.

**Figure 3:**
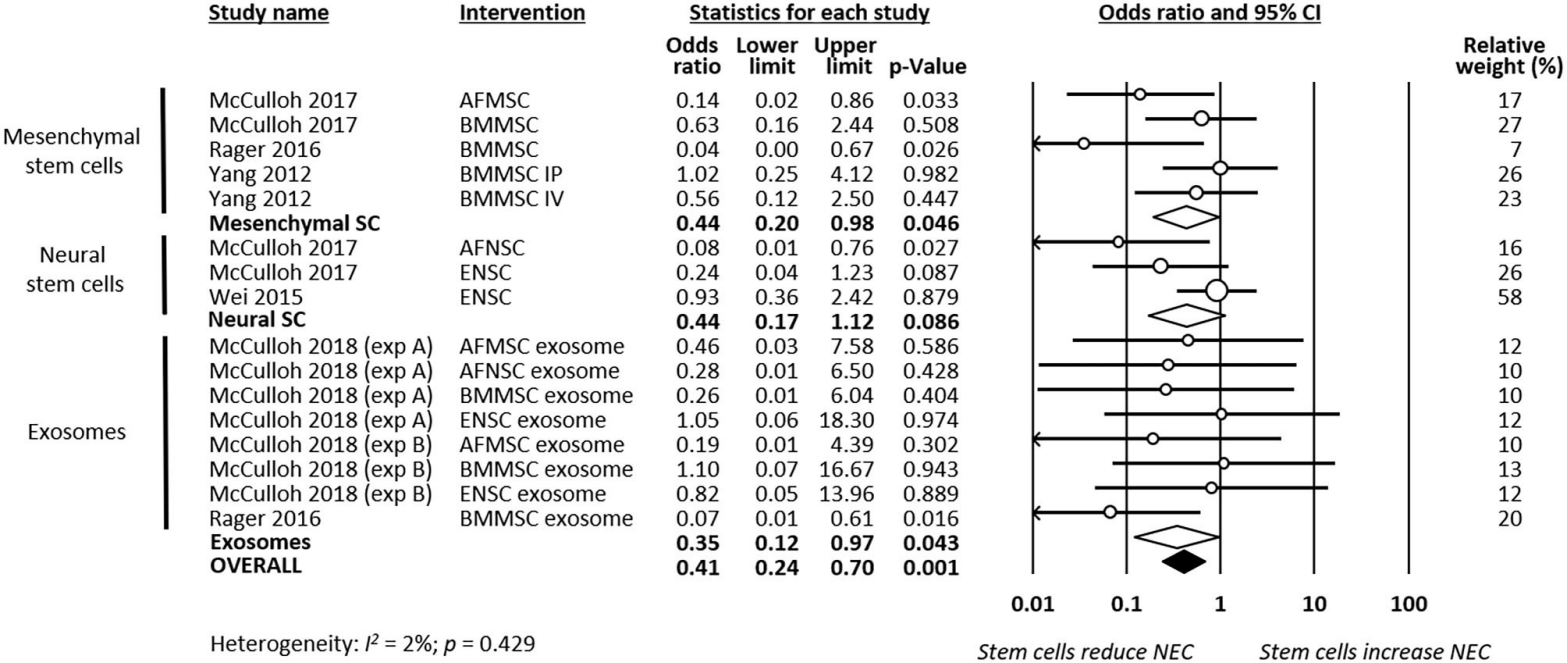
Meta-analysis on stem cells/stem cell-derived products and incidence of grade 2 necrotizing enterocolitis (NEC). AF, amniotic fluid; BM, bone marrow; ENSC, enteric neural stem cell; IP, intraperitoneal; IV, intravenous; MSC, mesenchymal stem cell; NSC, neural stem cell; SC, stem cell.

**Figure 4:**
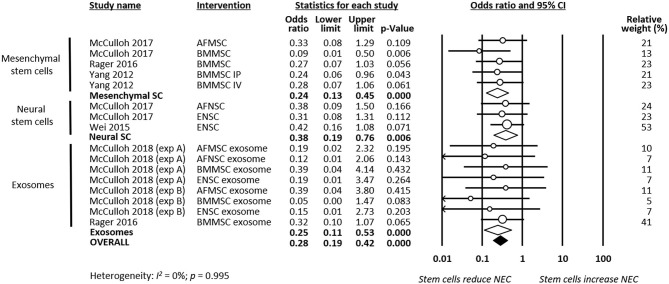
Meta-analysis on stem cells/stem cell-derived products and incidence of grade 3–4 necrotizing enterocolitis (NEC). AF, amniotic fluid; BM, bone marrow; ENSC, enteric neural stem cell; IP, intraperitoneal; IV, intravenous; MSC, mesenchymal stem cell; NSC, neural stem cell; SC, stem cell.

As shown in Figure 2, meta-analysis showed that stem cells and stem cell-derived products significantly reduced the incidence of any grade of NEC (*k* = 16, OR 0.22, 95% CI 0.16–0.32). Meta-analysis also found that stem cells and stem cell-derived products significantly reduced incidence of grade 2 NEC (*k* = 16, OR 0.41, 95% CI 0.24–0.70, Figure 3), as well as incidence of grade 3–4 NEC (*k* = 16, OR 0.28, 95% CI 0.19–0.42, Figure 4). As shown in Figures 2–4, the protective effects of stem cells were similarly observed in the subgroups of mesenchymal SC, neural SC, and SC-derived exosomes. Sensitivity analyses showed that the removal of the study that used mice instead of rats did not affect the significance of the results, nor did removal of experiments that used SC-derived exosomes (instead of SC) as the intervention (Supplementary Table 1).

#### Survival

**Figure 5:**
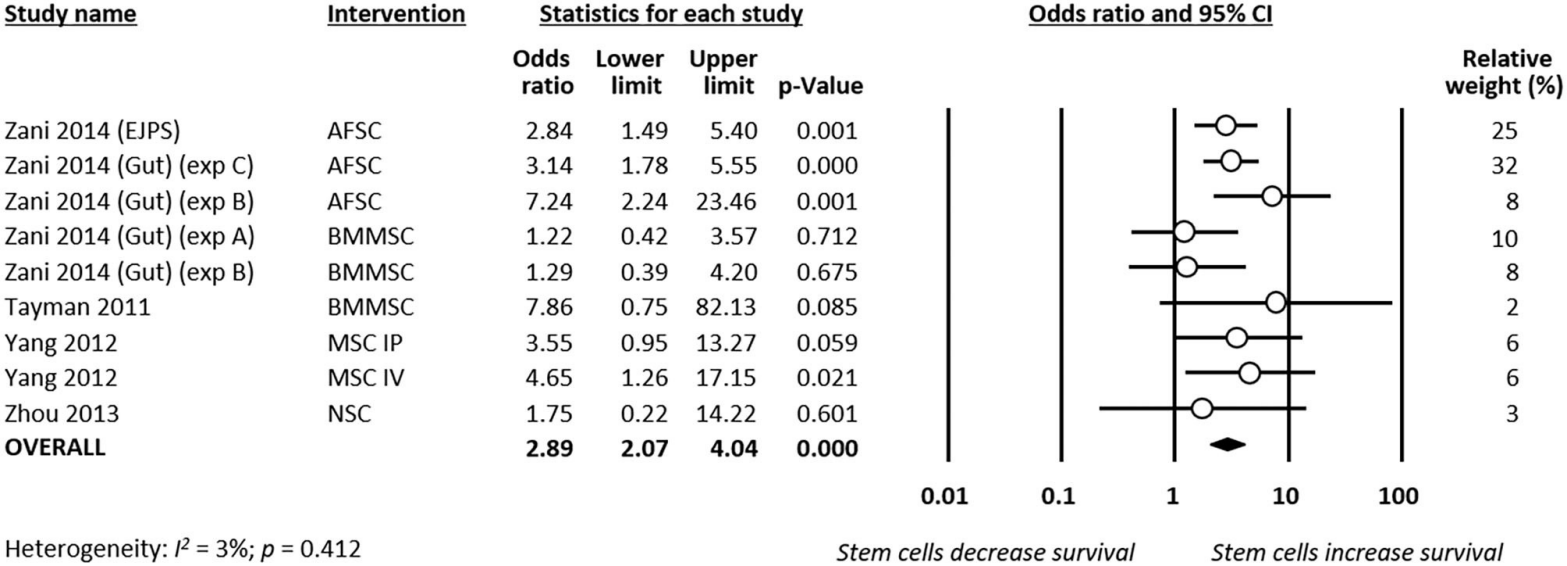
Meta-analysis on stem cells and survival at day 4 of life following necrotizing enterocolitis (NEC). AFSC, amniotic fluid stem cell; BMMSC, bone marrow mesenchymal stem cell; IP, intraperitoneal; IV, intravenous; MSC, mesenchymal stem cell; NSC, neural stem cell.

**Figure 6:**
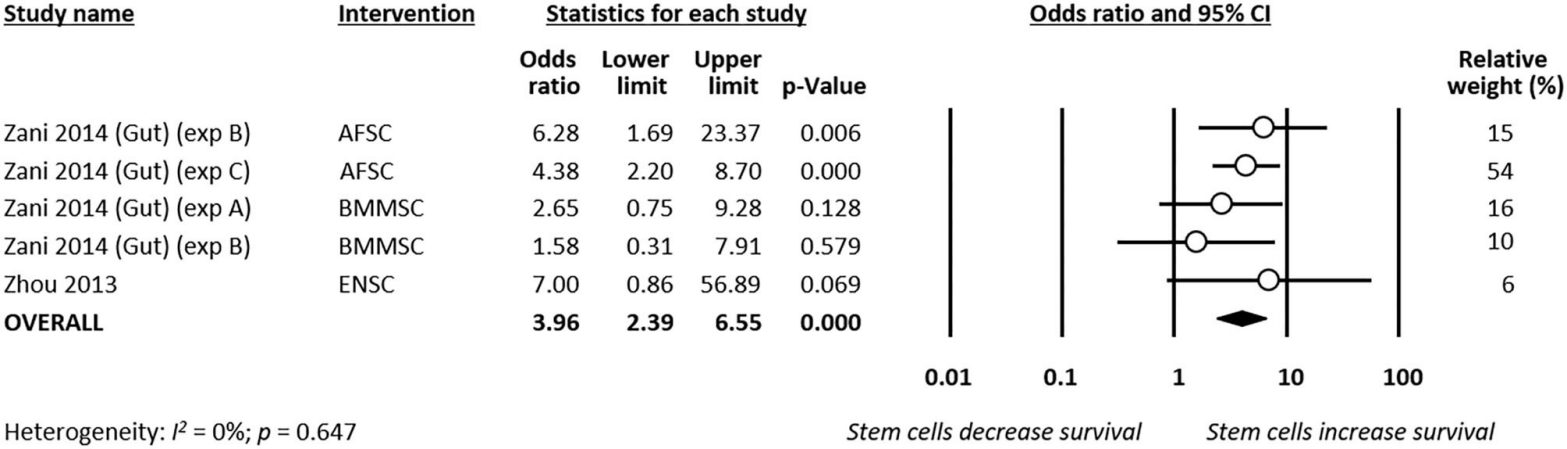
Meta-analysis on stem cells and survival at day 7 of life following necrotizing enterocolitis (NEC). AFSC, amniotic fluid stem cell; BMMSC, bone marrow mesenchymal stem cell; ENSC, enteric neural stem cell.

Meta-analysis showed that stem cells significantly improved survival at day 4 of life (*k* = 9, OR 2.89, 95% CI 2.07–4.04, Figure 5). Moreover, meta-analysis showed that stem cells significantly improved chance of survival at day 7 of life (*k* = 5, OR 3.96, 95% CI 2.39–6.55, Figure 6). Subgroup analysis based on the type of stem cells was not performed for this outcome due to the small number of studies in each subgroup.

### Meta-Analyses of Secondary Outcomes

**Figure 7:**
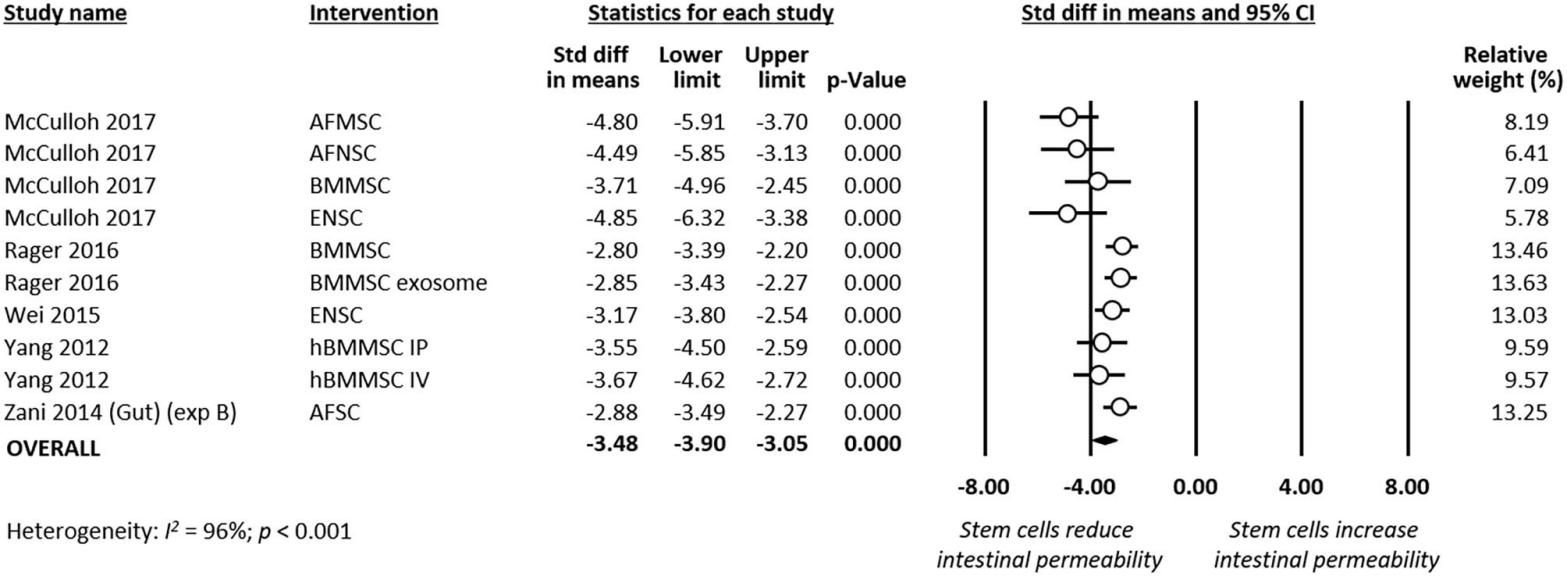
Meta-analysis on stem cells/stem cell-derived products and intestinal permeability following experimental necrotizing enterocolitis (NEC). AF, amniotic fluid; BM, bone marrow; ENSC, enteric neural stem cell; h, human; IP, intraperitoneal; IV, intravenous; MSC, mesenchymal stem cell; SC, stem cell.

Meta-analysis showed that stem cells and stem cell exosomes significantly reduced intestinal permeability (*k* = 10, SMD −3.48, 95% CI −3.90 to −3.05, Figure 7). Sensitivity analyses showed that the reduction remained significant after removing the sole experiment that used exosomes as the intervention and after removing the sole experiment that used mice as the experimental animal (Supplementary Table 1). Meta-analysis also found that stem cells improved intestinal motility (*k* = 2, SMD 3.73, 95% CI 3.09–4.38, Supplementary Figure 1) and reduced clinical sickness score (SMD −3.49, 95% CI −6.58 to −0.40, Supplementary Figure 2).

### Publication Bias

There were sufficient independent effect sizes (*k* > 10) to test two outcomes (any grade NEC and grade 3–4 NEC incidence) for publication bias. For any grade NEC (*k* = 16), the visual inspection and trim-and-fill analysis suggested funnel plot asymmetry (Supplementary Figure 3), but Egger's regression test was non-significant (*p* = 0.090). For the outcome NEC grade 3–4 (*k* = 16), the visual inspection and trim-and-fill analysis suggested funnel plot asymmetry (Supplementary Figure 4), and Egger's regression test supported the presence of significant publication bias (*p* < 0.001).

## Discussion

To the best of our knowledge, this is the first systematic review and meta-analysis of preclinical studies investigating the effects of stem cells in experimental NEC. Meta-analysis showed that stem cells and stem cell-derived exosomes increased survival and decreased both incidence and severity of histologically proven NEC in rodent models of the condition. The beneficial effects of stem cells were consistent despite the heterogeneity in the sort of cells or exosomes across the different studies.

The studies included in our review used bone marrow- and AF-derived MSCs, and AF- and fetal intestine-derived NSCs. Among non-embryonic stem cells, MSCs are considered to have a high therapeutic potential due to their ability of proliferation and multilineage differentiation (22, 53–55). MSCs are found in multiple tissues, including bone marrow, adipose tissue, placenta, chorion, amnion, umbilical cord, umbilical blood, and breast milk (22, 55). The data of the meta-analysis suggest that MSCs and NSCs have similar effects on experimental NEC. However, NSCs, compared with MSCs, are challenging to isolate and culture, potentially limiting their clinical utility (22). Two studies investigated the effects of AFSC (48, 49). AFSC are considered as an intermediate type between embryonic and adult stem cells, and they stained positively for a number of surface markers characteristic of MSCs and NSCs (51, 52). Interestingly, it has been shown that NSCs can be purified and derived from AFSC. As reviewed by McCulloch et al., these amniotic-fluid derived NSCs may have the ability to provide the same therapeutic effects than other NSCs but with the advantage of a greater easiness in obtaining them (52).

The results from this meta-analysis also suggest that exosomes are just as effective in reducing the incidence and severity of experimental NEC as the stem cells from which they derive. Accordingly, a previous meta-analysis showed that cell-free MSC-derived conditioned media had significant therapeutic effects in hyperoxic rodent models of bronchopulmonary dysplasia (21). Paracrine mediators such as stem cell-derived exosomes are emerging as a novel therapeutic strategy to overcome some of the limitations of stem cell therapy (56). Exosomes are small membrane vesicles of endocytic origin that exert their therapeutic actions by involving cell–cell interactions and transferring proteins, RNAs, and microRNAs (56). Interestingly, breast milk-derived exosomes, which are produced by a variety of cells, stimulate intestinal cell proliferation and differentiation (57, 58) and may be protective in experimental NEC (59).

The strengths of our systematic review included the breadth of the search strategy, the clear definition of inclusion and exclusion criteria, and the rigorous data evaluation and reporting by the use of international guidance and standards. However, the review has also a number of limitations that should be discussed. First, the included studies generally had small sample sizes that were not justified by power calculations. Second, evidence of publication bias was detected in some of the analyses (see supplementary Figure 4). Publication and other forms of reporting bias appear to occur in a greater proportion in preclinical than in clinical studies (60, 61). Therefore, studies with less positive results might not have been published. Third, a high number of the included studies came from the same research group (41–46). Fourth, although no statistical heterogeneity was detected, there was evident heterogeneity in the design of the studies, particularly in the type of stem cells (or exosomes) that were used. We conducted subgroup analyses to determine whether some of the stem cell types were more effective than others. However, these subgroups included a limited number of studies. Therefore, the results of the subgroup analysis should be interpreted with caution, and their main value is the generation of hypotheses for future research.

The fifth limitation to discuss was that the risk of bias assessment for the primary studies was hampered by low quality and/or completeness of reporting on relevant domains. This fact has already been highlighted in other systematic reviews of preclinical studies on neonatal pathological conditions (21, 62, 63). In general, studies blinded outcome assessment and had low risk of bias for selective outcome reporting. However, for all other elements of risk of bias, including sequence generation, randomization of intervention, comparability of baseline characteristics, allocation concealment, random housing, investigator blinding, random outcome assessment, and reporting bias, the information contained in the articles was insufficient to draw conclusions about risk of bias. It should be considered that the information may be incomplete because the authors considered some aspects of study design not sufficiently relevant to be mentioned (64). Despite the publication of extensive guidelines such as the ARRIVE guidelines (65), or the Gold Standard Publication Checklist (66), underreporting is a point of concern that harms the internal validity and the generalizability of the results (64). Therefore, it is imperative that studies report all characteristics of their animal, experimental, and intervention models (62, 64). This information, along with information regarding the experimental conditions, is especially important for future study comparisons and meta-analyses and, consequently, to optimize interventions for future translational and clinical studies (62, 64).

One final limitation that is inherent to all preclinical studies is the so-termed indirectness, which is defined as “how well the results translate from animals to the clinical situation” (28). Rodent models of NEC are frequently used by investigators because of their relative low costs and ease of breeding (14, 15). However, the inability to provide intensive care and parenteral nutrition to the pups limits the model to only a few days after NEC induction (14, 15). Moreover, it has been argued that the combination of insults (hypoxia, hypothermia, formula feeds, and bacterial products) that is used in rodent models is not what commonly leads to classical late-onset NEC in very and extremely preterm infants (63). In addition, the effects of an intervention may be species specific, making the extrapolation of experimental NEC to human preterm infants difficult (63). In this sense, it should be noted that some studies were carried out in term pups (see Table 1). Finally, the translational applicability of the studies included in this review is limited by the clinical difficulty of identifying those preterm infants who are starting to develop NEC and are therefore susceptible to early treatment with stem cells. Also related to translational applicability, the majority of the included studies administered the stem cells by intraperitoneal (IP) injection. IP administration of drugs in adults seems to be safe (67), but it is not known if this can be extrapolated to very preterm infants. Nevertheless, the IP route appears to be the most adequate for stem cell administration for the treatment of gastrointestinal disorders (68).

In conclusion, the data form this meta-analysis suggest that both stem cells and stem cell-derived exosomes prevented NEC in rodent experimental models. However, there is a need to explore this effect in other species and NEC models. Preclinical studies using animal models are invaluable tools for enriching our understanding of the etiopathogenesis and treatment of human diseases. Nevertheless, there is a need for greater homogeneity and clarity in both the experimental designs and in the way the design and results are reported in the publications. The participation of clinicians in the design of the experimental models would contribute to increase their translational applicability.

## Data Availability Statement

All datasets generated for this study are included in the article/Supplementary Material.

## Author Contributions

EV-M conceptualized the study, wrote the review protocol, carried out the systematic search, selected studies for inclusion, supervised data collection, carried out statistical analysis, and reviewed and revised the manuscript. TH carried out the systematic search, selected studies for inclusion, extracted study data, and drafted the first version of the manuscript. BK contributed to interpretation of results and reviewed and revised the manuscript. CH co-wrote the review protocol, supervised study inclusion and data collection, contributed to statistical analyses and interpretation of results, and reviewed and revised the manuscript. EV conceptualized and supervised the study, contributed to statistical analyses and interpretation of results, and reviewed and revised the manuscript. All authors contributed to the article and approved the submitted version.

## Conflict of Interest

The authors declare that the research was conducted in the absence of any commercial or financial relationships that could be construed as a potential conflict of interest.
