# Supplementary material for: Stem Cells as Therapy for Necrotizing Enterocolitis: A Systematic Review and Meta-Analysis of Preclinical Studies
Source: Front Pediatr. 2020 Dec 9;8:578984. doi: 10.3389/fped.2020.578984 (PMC7755993; doi:10.3389/fped.2020.578984)
Supplement: Supplementary file 1 [file Data_Sheet_1.PDF]

Stem cells as therapy for necrotizing enterocolitis: a systematic review and meta-analysis of preclinical studies

Eduardo Villamor-Martinez[1], Tamara Hundscheid[1], Boris W Kramer[1], Carlijn R Hooijmans[2], Eduardo Villamor[1*]

[1] Department of Pediatrics, Maastricht University Medical Center (MUMC+), School for Oncology and Developmental Biology (GROW), Maastricht, the Netherlands.

[2]Department for Health Evidence unit SYRCLE, Radboud University Medical Center, Nijmegen, The Netherlands.

*** Correspondence:**
Eduardo Villamor
e.villamor@mumc.nl

Supplementary Material

1 Supplementary Figures

Study name	Intervention	Std diff in means	Lower limit	Upper limit	p-Value	Relative weight (%)
Zani 2014 (Gut) (exp B)	AFSC	3.70	2.99	4.41	0.000	83
Zhou 2013	ENSC	3.90	2.33	5.47	0.000	17
**OVERALL**		**3.73**	**3.09**	**4.38**	**0.000**	

Statistics for each study

Heterogeneity: $I^2 = 0\%$; $p = 0.821$

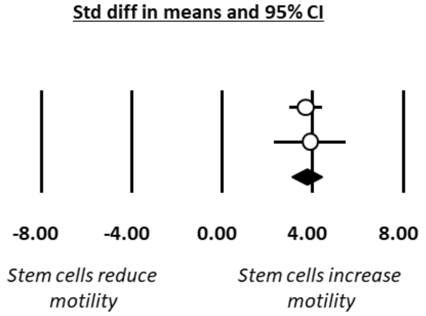

Supplementary Figure 1. Meta-analysis on stem cells and intestinal motility following necrotizing enterocolitis (NEC).
AFSC: amniotic fluid stem cells; ENSC: enteric neural stem cells.

Study name	Intervention	Std diff in means	Lower limit	Upper limit	p-Value	Relative weight (%)
Tayman 2011	BMMSC	-1.90	-2.87	-0.94	0.000	50
Zani 2014 (Gut) (exp B)	AFSC	-5.06	-5.94	-4.17	0.000	50
**OVERALL**		**-3.49**	**-6.58**	**-0.40**	**0.027**	

Statistics for each study

Heterogeneity: $I^2 = 96\%$; $p < 0.001$

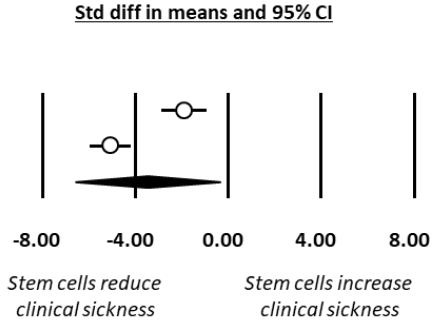

Supplementary Figure 2. Meta-analysis on stem cells and clinical sickness score following necrotizing enterocolitis (NEC).
AFSC: amniotic fluid stem cells; BMMSC: bone marrow mesenchymal stem cells.

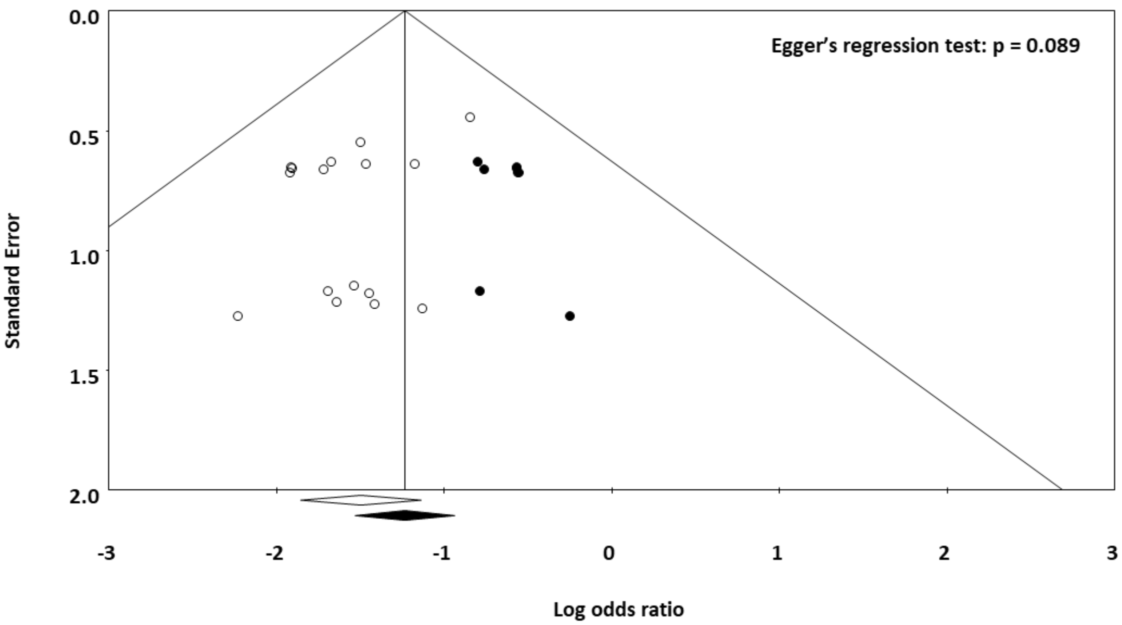

Supplementary Figure 3. Funnel plot of publication bias of the analysis on stem cells/stem cell-derived products and incidence of any grade necrotizing enterocolitis (NEC). The white dots represent the sample studies and the black dots represent the studies that have been added by the trim-and-fill.

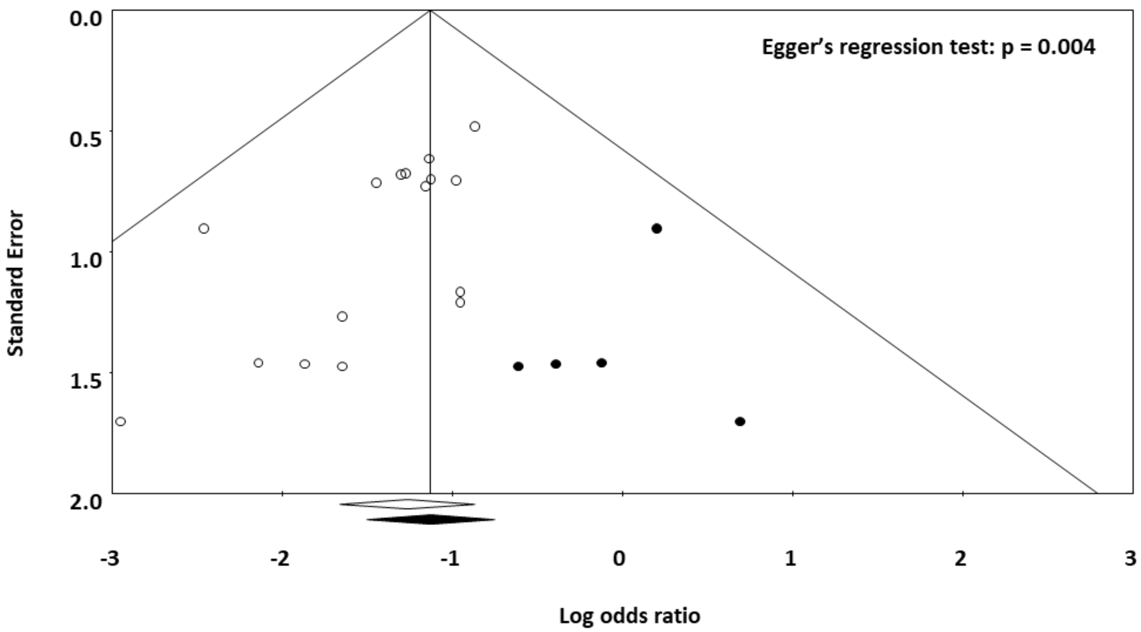

Supplementary Figure 4. Funnel plot of publication bias of the analysis on stem cells/stem cell-derived products and incidence of grade 3-4 necrotizing enterocolitis (NEC). The white dots represent the sample studies and the black dots represent the studies that have been added by the trim-and-fill.

2 Supplementary Tables

Supplementary Table 1. Sensitivity meta-analyses of stem cells and necrotizing enterocolitis, removing studies with non-rat animals and studies that use stem cell exosomes.

Outcome	Result original meta-analysis	95% CI	Sensitivity analysis	Result modified meta-analysis	95% CI
Any grade NEC	OR 0.22	0.16 to 0.32	Remove non-rat (i.e. mouse) study, Wei et al. 2015	OR 0.20	0.13 to 0.29
Grade 2 NEC	OR 0.44	0.27 to 0.71		OR 0.36	0.21 to 0.61
Grade 3-4 NEC	OR 0.28	0.19 to 0.42		OR 0.26	0.17 to 0.40
Intestinal permeability	SMD -3.48	-3.90 to -3.05		SMD -3.55	-4.05 to -3.05
Any grade NEC	OR 0.22	0.16 to 0.32	Remove interventions with stem cell exosomes	OR 0.23	0.15 to 0.35
Grade 2 NEC	OR 0.44	0.27 to 0.71		OR 0.33	0.15 to 0.71
Grade 3-4 NEC	OR 0.28	0.19 to 0.42		OR 0.30	0.19 to 0.47
Intestinal permeability	SMD -3.48	-3.90 to -3.05		SMD -3.59	-4.06 to -3.11

NEC: necrotizing enterocolitis.

3 Systematic search

PubMED search: 1100 hits

(Stem Cells [Mesh] OR] IPS cell*[tiab] OR hiPSC[tiab] OR hemangioblasts[tiab] OR hemogenic endothelial cell*[tiab] OR lymphoid progenitor cell*[tiab] OR lymphoid progenitor*[tiab] OR myeloid progenitor cell*[tiab] OR myeloid progenitor*[tiab] OR myoblast*[tiab] OR oligodendrocyte precursor cell*[tiab] OR blastomeres[tiab] OR embryoid bodies[tiab] OR germ cell*[tiab] OR amniotic fluid[Mesh] OR amniotic fluid*[tiab] OR

stem cells[tiab] OR stemcell*[tiab] OR Progenitor Cells[tiab] OR Progenitor Cell[tiab] OR Mother Cells[tiab] OR Mother Cell[tiab] OR Colony Forming Unit*[tiab]) OR CFU[tiab] OR multipotent cell* [tiab] OR precursor cell* [tiab] OR Mesenchymal Cell [tiab] OR MSCs [tiab] OR MSC [tiab] OR bone marrow derived cell*[tiab] OR BMSCs [tiab] OR BMSC [tiab] OR SC[tiab] OR SCs[tiab] OR Progenitor Cell* [tiab] OR Wharton Jelly[Mesh] OR Wharton Jelly Cell* [tiab] OR Wharton's Jelly Cell* [tiab] OR Wharton Jelly[tiab] OR Wharton's Jelly[tiab] OR ASC [tiab] OR ASCs [tiab] OR adipose stem cell* [tiab] OR pre-adipocyte* [tiab] OR fetal blood[mesh] OR foetal blood[tiab] OR fetus blood[tiab] OR foetus blood[tiab] OR umbilical cord blood [tiab] OR umbilical blood[tiab] OR fetal cord blood[tiab] OR foetal cord blood[tiab] OR fetus cord blood[tiab] OR foetus cord blood[tiab] OR UCB [tiab])

AND

(("enterocolitis, necrotizing"[MeSH Terms] OR Enterocolitis, Pseudomembranous[Mesh]OR NEC[tiab] OR Enterocolitis [tiab] OR Pneumatosis Intestinalis [tiab] OR Asphyxia Neonatorum [Mesh] or Asphyxia Neonatorum [tiab] OR newborn hypoxia[tiab] OR neonatal hypoxia[tiab]OR Necrotizing[tiab] OR necrotized[tiab] OR necrotised[tiab] OR necrotisation[tiab] OR necrotization[tiab] OR necrosis[tiab] OR Necrotize[tiab] OR Necrotising[tiab] OR Necrotise[tiab] OR Necroticans[tiab]) OR ((OR infan* [tiab] OR pup*[tiab]OR Infants, Newborn[Mesh] OR Newborn*[tiab] OR Neonate*[tiab] OR neonatal[tiab] OR Animal, Newborn[Mesh] OR Low Birth Weight*[tiab] OR gestational age[tiab] OR Decreased gestational age [tiab] OR premature gestation [tiab] OR prematurity [tiab] OR preterm[tiab] OR premature[tiab] OR postmature[tiab] OR Infant, Newborn, Diseases[Mesh] OR newborn disease*[tiab] OR infant disease*[tiab]) AND (intestinal injury[tiab] OR gut injury[tiab] OR "Gastrointestinal Diseases"[Mesh] OR gastrointestinal[tiab] OR Gastrointestinal Disease* [tiab] OR Cholera Infantum[tiab] OR Intestinal Disease* [tiab] OR intestinal inflammation[tiab] OR gut ischaemia[tiab] OR ischemia[tiab]OR enterocolitis ulcerosa necroticans[tiab] OR Enterocolitides[tiab] OR Pseudomembranous Enterocolitis[tiab] OR Pseudomembranous Enteritis[tiab] OR Pseudomembranous Colitis[tiab] OR Antibiotic-Associated Colitis[tiab] OR Antibiotic Associated Colitis[tiab] OR Clostridium Enterocolitis[tiab] OR Neutropenic Enterocolitis[tiab] OR typhlitis[tiab] OR Pneumatosis Cystoides Intestinalis[tiab]OR pneumatosis intestinalis[tiab] OR intestine pneumatosis[tiab] OR intestinal pneumatosis[tiab] OR Pneumoperitoneum [Mesh] OR Pneumoperitoneum[tiab] OR Mesenteric Ischemia[Mesh] OR Mesenteric Ischemia* [tiab] OR Mesenteric Ischaemia* [tiab] OR Mesenteric Vascular Insufficiency[tiab] OR Mesenteric Vascular Insufficiencies[tiab] OR Ulcer* [tiab] OR necrosis [tiab] OR necrotizing[tiab] OR necrotize[tiab] OR necrotising[tiab] OR necrotise[tiab])))

AND SYRCLE animal filter

EMBASE search: 530 hits

(exp Stem Cells/ OR Stem Cell.ti,ab,kw. OR stemcell*.ti,ab,kw. OR Progenitor Cells.ti,ab,kw. OR Progenitor Cell.ti,ab,kw. OR Mother Cells.ti,ab,kw. OR Mother Cell.ti,ab,kw. OR Colony Forming Unit*.ti,ab,kw. OR multipotent cell*.ti,ab,kw. OR precursor cell*.ti,ab,kw. OR exp Mesenchymal Stromal Cell/ OR stromal cell*.ti,ab,kw. OR MSCs.ti,ab,kw. OR MSC.ti,ab,kw. OR BMSCs.ti,ab,kw. OR BMSC.ti,ab,kw. OR SC.ti,ab,kw. OR SCs.ti,ab,kw. OR Progenitor Cell*.ti,ab,kw. OR exp Wharton jelly/ OR Wharton Jelly Cell*.ti,ab,kw. OR ASC.ti,ab,kw. OR ASCs.ti,ab,kw. OR exp fetal blood/ OR umbilical cord blood.ti,ab,kw. OR UCB.ti,ab,kw. OR exp **amnion fluid/**)

AND

((exp Necrotizing Enterocolitis/ OR NEC.ti,ab,kw. OR exp Enterocolitis/ OR Pneumatosis Intestinalis.ti,ab,kw. OR exp newborn hypoxia/ OR Necrotizing.ti,ab,kw. OR Necrotize.ti,ab,kw. OR Necrotising.ti,ab,kw. OR Necrotise.ti,ab,kw. OR Necroticans.ti,ab,kw.) OR ((exp prematurity/ OR exp newborn disease/ OR infan*.ti,ab,kw. OR Neonatal Prematurity.ti,ab,kw.OR Extremely Preterm Infant*.ti,ab,kw. OR Newborn*.ti,ab,kw. OR Neonate*.ti,ab,kw. OR neonatal.ti,ab,kw. OR exp Low Birth Weight/ OR Decreased gestational age.ti,ab,kw. OR premature gestation.ti,ab,kw. OR prematurity.ti,ab,kw.) AND (exp intestine injury/ OR intestinal injury.ti,ab,kw. OR gut injury.ti,ab,kw. OR gastrointestinal.ti,ab,kw. OR Gastrointestinal Disease*.ti,ab. OR Cholera Infantum.ti,ab,kw. OR Functional Gastrointestinal Disorder*.ti,ab,kw. OR Intestinal Disease*.ti,ab,kw. OR intestinal inflammation.ti,ab,kw. OR gut ischaemia.ti,ab,kw. OR enterocolitis ulcerosa necroticans.ti,ab,kw. OR Enterocolitides.ti,ab,kw. OR exp pseudomembranous colitis/ OR Pseudomembranous Enterocolitis.ti,ab,kw. OR Pseudomembranous Enteritis.ti,ab,kw. OR Pseudomembranous Colitis.ti,ab,kw. OR Antibiotic Associated Colitis.ti,ab,kw. OR Antibiotic Associated Colitis.ti,ab,kw. OR Clostridium Enterocolitis.ti,ab,kw. OR exp neutropenic enterocolitis/ OR Neutropenic Enterocolitis.ti,ab,kw. OR typhlitis.ti,ab,kw. OR exp Pneumatosis Intestinalis/ OR Pneumatosis Intestinalis.ti,ab,kw. OR pneumatosis intestinalis.ti,ab,kw. OR exp Pneumoperitoneum/ OR exp Mesenteric Ischemia/ OR Mesenteric Ischemia*.ti,ab,kw. OR mesentery ischemia*.ti,ab,kw. OR Mesenteric Vascular Insufficiency.ti,ab,kw. OR Mesenteric Vascular Insufficiencies.ti,ab,kw. OR Ulcer*.ti,ab,kw.)))

AND **SYRCLE animal filter:** (exp animal experiment/ or exp animal model/ or exp experimental animal/ or exp transgenic animal/ or exp male animal/ or exp female animal/ or exp juvenile animal/ OR animal/ OR chordata/ OR vertebrate/ OR tetrapod/ OR exp fish/ OR amniote/ OR exp amphibia/ OR mammal/ OR exp reptile/ OR exp sauropsid/ OR therian/OR exp monotremate/ OR placental mammals/ OR exp marsupial/ OR Euarchontoglires/ OR exp Afrotheria/ OR exp Boreoeutheria/ OR exp Laurasiatheria/ OR exp Xenarthra/ OR primate/ OR exp Dermoptera/ OR exp Glires/ OR exp Scandentia/ OR Haplorhini/ OR exp prosimian/ OR simian/ OR exp tarsiiform/ OR Catarrhini/ OR exp Platyrrhini/ OR ape/ OR exp Cercopithecidae/ OR hominid/ OR exp hylobatidae/ OR exp chimpanzee/ OR exp gorilla/ OR exp orang utan/ OR (animal OR animals OR pisces OR fish OR fishes OR catfish OR catfishes OR sheatfish OR silurus OR arius OR heteropneustes OR clarias OR gariepinus OR fathead minnow OR fathead minnows OR pimephales OR promelas OR cichlidae OR trout OR trouts OR char OR chars OR salvelinus OR salmo OR oncorhynchus OR guppy OR guppies OR millionfish OR poecilia OR goldfish OR goldfishes OR carassius OR auratus OR mullet OR mullets OR mugil OR curema OR shark OR sharks OR cod OR cods OR gadus OR morhua OR carp OR carps OR cyprinus OR carpio OR killifish OR eel OR eels OR anguilla OR zander OR sander OR lucioperca OR stizostedion OR turbot OR turbots OR

psetta OR flatfish OR flatfishes OR plaice OR pleuronectes OR platessa OR tilapia OR tilapias OR oreochromis OR sarotherodon OR common sole OR dover sole OR solea OR zebrafish OR zebrafishes OR danio OR rerio OR seabass OR dicentrarchus OR labrax OR morone OR lamprey OR lampreys OR petromyzon OR pumpkinseed OR pumpkinseeds OR lepomis OR gibbosus OR herring OR clupea OR harengus OR amphibia OR amphibian OR amphibians OR anura OR salientia OR frog OR frogs OR rana OR toad OR toads OR bufo OR xenopus OR laevis OR bombina OR epidalea OR calamita OR salamander OR salamanders OR newt OR newts OR triturus OR reptilia OR reptile OR reptiles OR bearded dragon OR pogona OR vitticeps OR iguana OR iguanas OR lizard OR lizards OR anguis fragilis OR turtle OR turtles OR snakes OR snake OR aves OR bird OR birds OR quail OR quails OR coturnix OR bobwhite OR colinus OR virginianus OR poultry OR poultries OR fowl OR fowls OR chicken OR chickens OR gallus OR zebra finch OR taeniopygia OR guttata OR canary OR canaries OR serinus OR canaria OR parakeet OR parakeets OR grasskeet OR parrot OR parrots OR psittacine OR psittacines OR shelduck OR tadorna OR goose OR geese OR branta OR leucopsis OR woodlark OR lullula OR flycatcher OR ficedula OR hypoleuca OR dove OR doves OR geopelia OR cuneata OR duck OR ducks OR greylag OR graylag OR anser OR harrier OR circus pygargus OR red knot OR great knot OR calidris OR canutus OR godwit OR limosa OR lapponica OR meleagris OR gallopavo OR jackdaw OR corvus OR monedula OR ruff OR philomachus OR pugnax OR lapwing OR peewit OR plover OR vanellus OR swan OR cygnus OR columbianus OR bewickii OR gull OR chroicocephalus OR ridibundus OR albifrons OR great tit OR parus OR aythya OR fuligula OR streptopelia OR risoria OR spoonbill OR platalea OR leucorodia OR blackbird OR turdus OR merula OR blue tit OR cyanistes OR pigeon OR pigeons OR columba OR pintail OR anas OR starling OR sturnus OR owl OR athene noctua OR pochard OR ferina OR cockatiel OR nymphicus OR hollandicus OR skylark OR alauda OR tern OR sterna OR teal OR crecca OR oystercatcher OR haematopus OR ostralegus OR shrew OR shrews OR sorex OR araneus OR crocidura OR russula OR european mole OR talpa OR chiroptera OR bat OR bats OR eptesicus OR serotinus OR myotis OR dasycneme OR daubentonii OR pipistrelle OR pipistrellus OR cat OR cats OR felis OR catus OR feline OR dog OR dogs OR canis OR canine OR canines OR otter OR otters OR lutra OR badger OR badgers OR meles OR fitchew OR fitch OR foumart or foulmart OR ferrets OR ferret OR polecat OR polecats OR mustela OR putorius OR weasel OR weasels OR fox OR foxes OR vulpes OR common seal OR phoca OR vitulina OR grey seal OR halichoerus OR horse OR horses OR equus OR equine OR equidae OR donkey OR donkeys OR mule OR mules OR pig OR pigs OR swine OR swines OR hog OR hogs OR boar OR boars OR porcine OR piglet OR piglets OR sus OR scrofa OR llama OR llamas OR lama OR glama OR deer OR deers OR cervus OR elaphus OR cow OR cows OR bos taurus OR bos indicus OR bovine OR bull OR bulls OR cattle OR bison OR bisons OR sheep OR sheeps OR ovis aries OR ovine OR lamb OR lambs OR mouflon OR mouflons OR goat OR goats OR capra OR caprine OR chamois OR rupicapra OR leporidae OR lagomorpha OR lagomorph OR rabbit OR rabbits OR oryctolagus OR cuniculus OR laprine OR hares OR lepus OR rodentia OR rodent OR rodents OR murinae OR mouse OR mice OR mus OR musculus OR murine OR woodmouse OR apodemus OR rat OR rats OR rattus OR norvegicus OR guinea pig OR guinea pigs OR cavia OR porcellus OR hamster OR hamsters OR mesocricetus OR cricetulus OR cricetus OR gerbil OR gerbils OR jird OR jirds OR meriones OR unguiculatus OR jerboa OR jerboas OR jaculus OR chinchilla OR chinchillas OR beaver OR beavers OR castor fiber OR castor canadensis OR sciuridae OR squirrel OR squirrels OR sciurus OR chipmunk OR chipmunks OR marmot OR marmots OR marmota OR suslik OR susliks OR spermophilus OR cynomys OR cottonrat OR cottonrats OR sigmodon OR vole OR voles OR microtus OR myodes OR glareolus OR primate OR primates OR prosimian OR prosimians OR lemur OR lemurs OR lemuridae OR loris OR bush baby OR bush babies OR bushbaby OR bushbabies OR galago OR galagos OR anthropoidea

OR anthropoids OR simian OR simians OR monkey OR monkeys OR marmoset OR marmosets OR callithrix OR cebuella OR tamarin OR tamarins OR saguinus OR leontopithecus OR squirrel monkey OR squirrel monkeys OR saimiri OR night monkey OR night monkeys OR owl monkey OR owl monkeys OR douroucoulis OR aotus OR spider monkey OR spider monkeys OR ateles OR baboon OR baboons OR papio OR rhesus monkey OR macaque OR macaca OR mulatta OR cynomolgus OR fascicularis OR green monkey OR green monkeys OR chlorocebus OR vervet OR vervets OR pygerythrus OR hominoidea OR ape OR apes OR hylobatidae OR gibbon OR gibbons OR siamang OR siamangs OR nomascus OR symphalangus OR hominidae OR orangutan OR orangutans OR pongo OR chimpanzee OR chimpanzees OR pan troglodytes OR bonobo OR bonobos OR pan paniscus OR gorilla OR gorillas OR troglodytes).ti,ab.)
